# B cells and the therapeutic index of checkpoint blockade

**DOI:** 10.1172/JCI212237

**Published:** 2026-11-02

**Authors:** Chuan-Yuan Li

**Affiliations:** 1 Chinese Institute for Molecular and Cellular Therapeutics, Chinese Institutes for Medical Research, Beijing, China.; 2 School of Basic Medical Sciences, Capital Medical University, Beijing, China.

## Abstract

Combined CTLA-4 and PD-1 blockade is effective in treating melanoma and additional cancers but frequently causes immune-related adverse events (irAEs). irAEs cause treatment disruption and discontinuation and remain difficult to manage clinically. In this issue, Dhodapkar and colleagues assessed data from a small randomized clinical trial testing rituximab-mediated B cell depletion in combined CTLA-4/PD-1 blockade–treated patients with advanced melanoma. Despite premature discontinuation of the trial due to a high rate of rituximab hypersensitivity, they report that preemptive B cell depletion was associated with fewer severe toxicities without evident loss of antitumor activity. These findings raise broader questions about the organization of checkpoint-induced autoimmunity, the emerging concept of immune reset, and the compartment-specific roles of B cells in antitumor immunity.

Immune checkpoint blockade (ICB) has transformed the treatment of melanoma and other cancers (1), but its success is limited by immune-related adverse events (irAEs). With combined ipilimumab (anti-CTLA-4) and nivolumab (anti-PD-1) therapy, the incidence of grade 3 or higher toxicity approaches 60% (2), and management remains largely reactive. This has reinforced the view that efficacy and toxicity are tightly linked consequences of immune activation. In this issue of the JCI, Dhodapkar and colleagues tested whether B cell manipulation might instead widen the therapeutic index of ICB (3).

In their randomized pilot study, 14 patients with advanced melanoma received ipilimumab plus nivolumab with or without one cycle of rituximab, an anti-CD20 mAb that selectively depletes B cells. Grade 3 or higher irAEs occurred in 4 of 7 patients in the control group and 1 of 7 patients who had been treated with rituximab, while objective responses appeared similar. The study closed early because of frequent rituximab hypersensitivity, including grade 3 reactions in 3 of 7 patients. Although far too small to establish clinical efficacy or safety, the randomized design and accompanying immune profiling provide a useful experiment for asking whether B cell–dependent toxicity circuits can be modified without broadly suppressing antitumor immunity.

## Toxicity may depend on immune organization

The most instructive observation may be what B cell depletion did not do. Rituximab did not induce broad immunologic quiescence. ICB in the depleted setting was accompanied by increased CXCL13^+^ ICOS^+^ CD4^+^ T cells and cytolytic CD8^+^ T cell populations, and enhanced inflammatory and NF-κB–associated programs in monocytes (3). Severe immune toxicity therefore declined despite substantial — and in some compartments greater — immune activation.

This complements earlier work showing that B cell changes can precede clinical autoimmunity after combined ICB and that interactions among peripheral helper-like CD4^+^ T cells, B cells, and regulatory T cells may contribute to irAEs (4, 5). In the current study, analysis of pretreatment B cells from patients who subsequently developed severe irAEs revealed a distinct crosstalk program enriched in activated naive B cells, with supporting correlation in an independent melanoma cohort (3, 6, 7). Given the small number of patients, these associations are best viewed as hypothesis-generating and require validation in larger cohorts.

Together, Dhodapkar et al.’s data suggest that the development of irAEs may depend not only on how much immunity is generated, but on how immune activation is organized. B cells can present antigen, provide costimulation, produce cytokines, and shape T cell differentiation. Removing a relevant B cell population could therefore alter the cellular partners available to ICB-activated T cells without necessarily diminishing T cell activation itself. ICB toxicity may thus reflect a pathogenic immune configuration rather than simply excessive immune activity (Figure 1).

## Interpreting the clinical signal

Several features should shape interpretation of Dhodapkar et al.’s study. Rituximab hypersensitivity may prompt corticosteroid treatment, treatment delays, or changes in checkpoint exposure, all of which could influence subsequent toxicity and should be carefully examined in future studies. Baseline imbalances are also unsurprising in seven-patient treatment arms and preclude interpretation of apparent progression-free survival differences. Inflammatory myeloid signatures measured soon after treatment may likewise reflect more than one biological process, including recent infusion reactions. These considerations do not negate the observed signal, but reinforce the need for confirmation in a larger trial with careful accounting of concomitant medications and treatment exposure.

## Prevention may differ from immune reset

The study is timely because B cell depletion is being reconsidered in autoimmune disease. Obinutuzumab, a humanized anti-CD20 mAb, has shown efficacy in lupus nephritis (8), while CD19-directed CAR T cell therapy has produced drug-free remissions in severe refractory autoimmune diseases that can persist after B cell reconstitution (9). These observations have contributed to the concept of an “immune reset,” which posits that a time-limited intervention may dismantle an established pathogenic network and permit immune reconstitution without simply recreating the previous autoimmune state (10, 11).

The present study does not demonstrate such a reset. The clonality, autoreactivity, and functional properties of reconstituted B cells were not examined, and most high-grade toxicities from ipilimumab/nivolumab arise during or shortly after induction, at timepoints overlapping with B cell depletion (2). Persistent irAE-free survival after B cell recovery therefore does not by itself establish a reset mechanism.

The analogy nevertheless suggests a useful distinction. In established autoimmune disease, memory populations, antibody-producing cells, and tissue immune networks are already present, and depth of depletion may influence durable remission. At the initiation of ICB, by contrast, the pathogenic network may not yet be fully assembled. Preventing such a network from forming may require less than dismantling one that is established. If so, timing and cellular selectivity could be as important as depth of depletion.

## The B cell paradox

Any model assigning a pathogenic role to B cells in ICB must also account for evidence that intratumoral B cells and tertiary lymphoid structures (TLSs) are associated with favorable responses to ICB in melanoma and other tumors (12–14). These observations need not be contradictory. B cell function likely depends strongly on differentiation state, anatomical compartment, and cellular partners. Circulating activated B cells involved in potentially autoreactive T–B interactions may behave differently from antigen-experienced B cells within organized tumor-associated TLSs.

The increase in CXCL13^+^ ICOS^+^ CD4^+^ T cells after rituximab is interesting in this regard, because CXCL13-producing helper populations have been linked to productive antitumor immunity (15). It suggests that checkpoint-induced helper programs can remain active even as circulating B cells are depleted. The key determining factor may be the cellular or tissue context in which those programs operate.

The current study cannot determine whether anti-CD20 therapy preserved, impaired, or remodeled tumor-associated B cell niches. Anti-CD20 treatment may affect circulating and tissue B cell compartments differently and is known to spare CD20-negative plasma cells (10); here, plasmablasts were relatively enriched among residual B cells after depletion (3). In subsequent studies, paired tumor sampling and spatial profiling will therefore be important. For now, TLS biology is best viewed as an important counterpoint that underscores the compartmentalized nature of B cell function.

## From perturbation to clinical strategy

Rituximab as administered here is not ready for routine prophylaxis against ICB toxicity; the hypersensitivity signal alone argues for caution. The greater value of rituximab in the context of ICB irAEs may be as a biological perturbation identifying B cell–dependent circuitry as a potentially tractable intervention target.

The next questions are mechanistic and translational. Are activated naive B cells drivers of irAEs or markers of an autoimmune-prone state? Which B cell functions are indispensable? Is there a discrete window during which transient intervention can alter the trajectory of checkpoint immunity? Can peripheral pathogenic interactions be disrupted while preserving beneficial tumor-associated B cell responses? The pretreatment crosstalk program that Dhodapkar et al. identified in patients who went on to develop severe irAEs also raises the possibility of biomarker-guided prophylaxis, reserving intervention for patients with a high-risk immune state.

The unexpectedly high frequency of rituximab hypersensitivity raises a separate question: might CTLA-4 and PD-1 blockade lower the threshold for drug hypersensitivity in humans? This concept remains speculative, but it is testable and may have implications beyond oncology (16).

The study by Dhodapkar and colleagues should therefore be read less as a prescription for rituximab than as evidence that the relationship between ICB efficacy and toxicity may be experimentally tractable. The therapeutic index of ICB may depend not only on the strength of immune activation, but also on which cells interact, where those interactions occur, and when the relevant networks form. A trial closed because of toxicity may thus have generated two useful hypotheses: that selected B cell–dependent circuits contribute to irAEs, and that checkpoint pathways may also restrain drug hypersensitivity. Both point toward a broader goal: increasing the success of ICB depends on not simply releasing immune brakes, but on learning how to steer the response once they are released.

## Manuscript information

### AI disclosure.

AI was used to revise the manuscript text and in the drafting of Figure 1.

### Conflict of interest.

CYL is an inventor on patents US12275986B2, US12558351B2, and US20250084139A1.

## Figures and Tables

**Figure 1:**
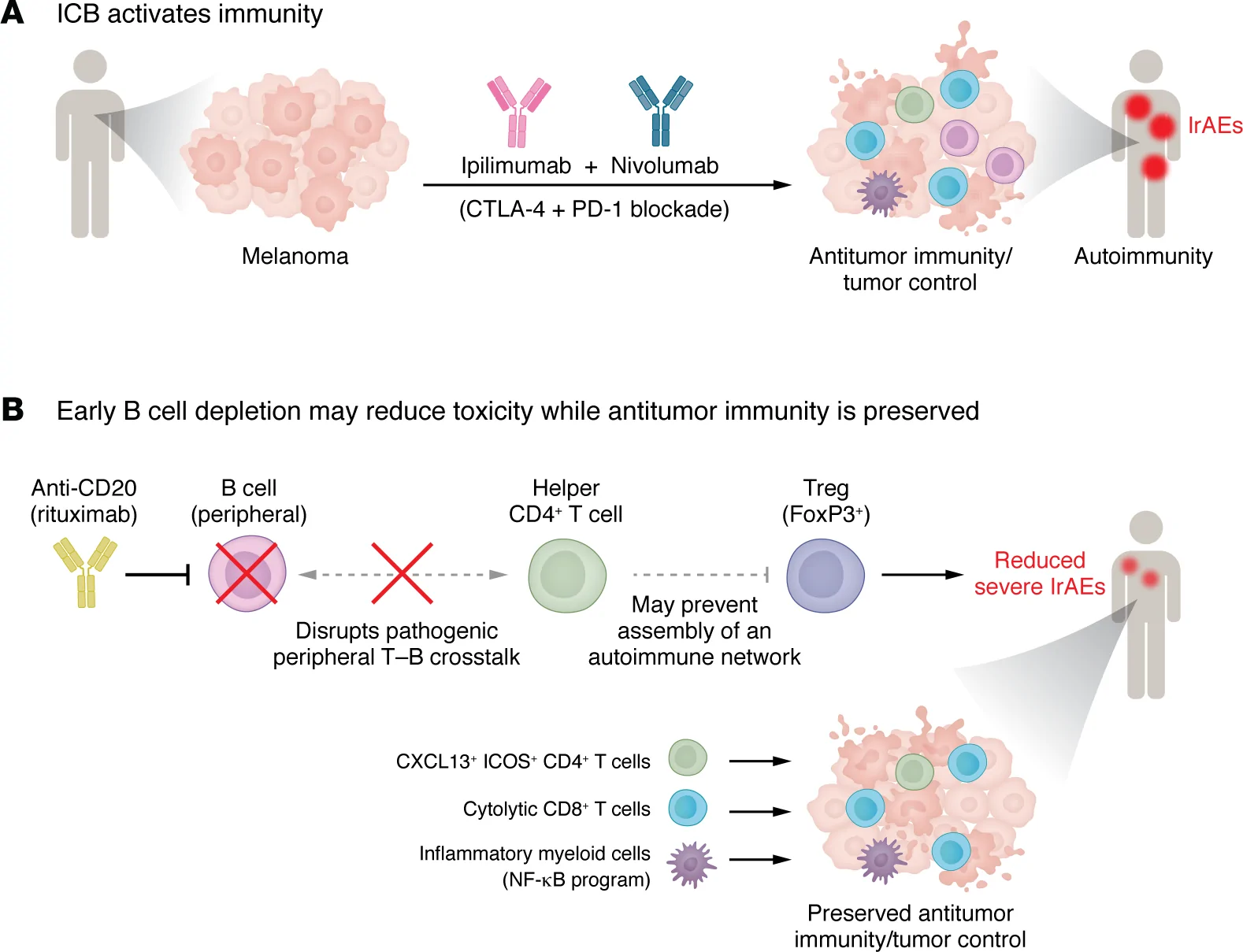
B cell depletion may alter the organization and timing of ICB-induced immunity. (**A**) Combined CTLA-4 and PD-1 blockade activates antitumor immunity but can also promote immune-related adverse events (irAEs). (**B**) Dhodapkar et al. (3) showed that transient early B cell depletion with anti-CD20 therapy may disrupt pathogenic peripheral T–B cell crosstalk and thereby reduce the likelihood that a self-sustaining autoimmune network becomes established. Importantly, this intervention may not require broad suppression of ICB-induced immunity: CXCL13^+^ICOS^+^ CD4^+^ T cells, cytolytic CD8^+^ T cells, and inflammatory myeloid programs can remain active, potentially preserving antitumor immunity and tumor control. This model differs from the emerging “immune reset” paradigm in established autoimmune disease, in which deeper B cell depletion may be required to dismantle an existing pathogenic network. In the ICB setting, a more limited and temporally targeted intervention may instead prevent an autoimmune state from being established. Whether systemic B cell depletion alters beneficial intratumoral B cells or tertiary lymphoid structures remains an important unresolved question.

## Version 1 11/02/2026

Electronic publication
